# Intranasal Human Growth Hormone (hGH) Induces IGF-1 Levels Comparable With Subcutaneous Injection With Lower Systemic Exposure to hGH in Healthy Volunteers

**DOI:** 10.1210/jc.2014-4146

**Published:** 2015-10-01

**Authors:** Andrew L. Lewis, Faron Jordan, Tina Patel, Kirk Jeffery, Gareth King, Martin Savage, Stephen Shalet, Lisbeth Illum

**Affiliations:** Critical Pharmaceuticals Ltd (A.L.L., F.J., T.P., K.J., G.K., L.I.), BioCity Nottingham, Nottingham NG1 1GF, United Kingdom; Department of Endocrinology (M.S.), William Harvey Research Institute, Barts and the Royal London School of Medicine and Dentistry, London E1 1BB, United Kingdom; and Department of Endocrinology (S.S.), Christie Hospital, Manchester M20 4BX, United Kingdom

## Abstract

**Context:**

The development of an improved, efficacious human GH (hGH) product administered by a noninjectable route of delivery such as the nasal route is highly desirable. We have developed a novel nasal hGH product (CP024) that showed excellent nasal absorption in animal models; however, the translation of these results into the clinical setting is essential because past attempts to develop such formulations by other groups have been unable to induce IGF-1 in man.

**Objective:**

The objective of the study was to assess the pharmacokinetics, pharmacodynamics, and tolerability of CP024 compared with a sc hGH injection.

**Design:**

This was a single-center, nonrandomized placebo-controlled, open-label, five-way crossover study in eight healthy volunteers.

**Setting:**

The study was carried out at a contract research organization, Quotient Bioresearch.

**Volunteers:**

Eight healthy male volunteers, given an iv infusion of octreotide to suppress the endogenous GH secretion during the study period, participated in the study. No volunteers were withdrawn due to side effects.

**Main Outcome Measures:**

Measurement of hGH and IGF-1 levels and tolerability of the drug product was performed.

**Results:**

No serious adverse events were reported and no subjects withdrawn from study due to the treatment. After the nasal administration of CP024, 3-fold higher hGH blood levels were obtained as compared with hGH nasal control. The relative bioavailability was about 3%. CP024 (given twice daily) induced a significant increase in IGF-1 levels up to 19 hours after administration, with no significant difference to those obtained after the sc injection of hGH.

**Conclusions:**

The study indicates that CP024 is a promising candidate for an efficacious nasal product for the treatment of GH deficiency due to induction of IGF-1 similar to that after a sc injection, despite the lower plasma hGH concentration obtained. A dose-response study is needed to evaluate the optimal nasal dose.

When human GH (hGH), isolated from the pituitaries of human cadavers, was first used to treat GH deficiency in children, it was injected im three times a week, primarily due to the scarcity of supply (1). The availability of recombinant hGH in the mid-1980s not only avoided the iatrogenic risk of transmission of Creutzfeldt-Jakob disease but also allowed an increase in production of the hormone and hence made possible the investigation of alternative treatment regimens. Because sc injections are less painful than im injections and are easier to self-administer, this became the preferred route of administration in children and adults, eventually by means of the easy-to-use pen-type delivery devices (eg, Novo Nordisk Norditropin FlexPro Pen; Eli Lilly Humatrope Pen). Despite having a very different pharmacokinetic profile to that found after im injection, the efficacy after the sc administration of hGH was shown to have increased, and interestingly, neither route of administration produces a pharmacokinetic profile equivalent to the endogenous hGH profile in healthy volunteers (2, 3). Since that time, with the aim of developing formulations that might further increase efficacy, increase patient compliance and persistence, or reduce side effects, investigators have evaluated the administration of hGH via the iv, oral, pulmonary, transdermal, and nasal routes (2, 4–8). Also, numerous sustained-release formulations have been investigated, with two reaching the market (9, 10). Despite these efforts and the significant advances achieved in drug delivery over the last few decades, hGH replacement therapy still requires administration via injection, usually on a daily basis.

Numerous studies have shown that patients (particularly children) and their caregivers dislike the administration of injections due to needle phobia, the (real or anticipated) pain associated with them, the inconvenience and/or complexity of the regimen (eg, the multiple steps involved in the injection protocol and storage and needle disposal issues) as well as injection site reactions such as bruising (11, 12). Indeed, in the case of pediatric GH deficiency treatment, adherence has been estimated to be as low as 18% (13–15). As might be expected, adherence to hGH replacement therapy is correlated to efficacy, and missing as little as one injection per week has been shown to significantly reduce the growth rate in children (15, 16). Not only is the health care benefit reduced by nonadherence, but also this results in increased health care costs as physicians use additional diagnostic procedures and alter the treatment to achieve therapeutic success and/or treat the consequences of GH deficiency, eg, increase the dose, prescribe additional drugs, and/or try alternative therapeutic interventions (13, 15). The development of improved efficacious hGH products administered via alternative routes of delivery is therefore highly desirable.

The nasal route of drug delivery is particularly attractive due to the favorable environment in the nasal cavity for drug absorption (the pH and paucity of enzymes, its abundant blood supply, and absorptive mucosa) and the relative ease with which nasal sprays can be administered (17). A recent study showed that 69% of the children participating in the study preferred the nasal route of administration of an influenza vaccine over injection (18), and a follow-up study showed that parents and caregivers would also prefer the nasal route as long as efficacy was maintained (19).

The pharmacokinetics of nasal administration of hGH has been evaluated in volunteers and in patients using absorption enhancers to improve absorption across the nasal mucosa (20–22). However, pharmacodynamic studies in patients using nasally administered hGH did not induce increased levels of IGF-1, and hence, these formulations were considered therapeutically inactive (4).

We recently discovered a novel absorption enhancer, CriticalSorb (Solutol HS15, BASF), that promotes the transepithelial transport of coformulated proteins and peptides across the nasal membrane. This resulted in the development of a dry powder nasal formulation of hGH (CP024) (23). A variety of species has been shown to express GH and GH receptors in multiple tissues in the central nervous system such as the thalamus, hypothalamus, pituitary, and hippocampus, which implicates a role for GH signaling in the development and maintenance of neural tissue as well as cognition and memory (24) Furthermore, the regulation of GH synthesis and secretion is complex and involves modulation by both central and peripherally derived modulators (25) We hypothesized that the proximity of the pituitary gland and its blood supply to the site of absorption, the potential for direct nose to brain delivery of hGH with the possibly of reaching hGH receptors in the brain, and the potential for a more physiologically equivalent hGH plasma profile (26, 27) could make the nasal route of delivery particularly promising for GH replacement therapy. After the completion of a preclinical safety assessment package on both CriticalSorb and CP024, the current studies evaluated the safety and tolerability, pharmacokinetics, and pharmacodynamics of single and multiple doses of CP024 in healthy volunteers.

## Materials and Methods

Recombinant hGH was supplied by Sandoz. hGH from the same supplier was also used to manufacture Omnitrope. Omnitrope and octreotide (Sun Pharmaceuticals) were supplied through a local hospital pharmacy. CP024 (a spray dried nasal powder formulation comprising hGH, Solutol HS 15, a gelling agent, and other excipients) was manufactured for Critical Pharmaceuticals by Quotient Clinical. The spray dried powder was filled into nasal Aptar UDS powder devices (Aptar Pharma).

### Study design

The study was a single-center, nonrandomized, placebo-controlled, open-label, five-way crossover study in eight healthy male volunteers designed to assess the pharmacokinetics, pharmacodynamics, and tolerability of the CP024 formulation (5 mg hGH in each nostril) compared with a sc injection of 1 mg Omnitrope and 5 mg hGH administered in each nostril formulated as a simple solution. Furthermore, the effect of a dosing schedule as with either one or two daily doses was also investigated.

The following five regimens were administered:

- Regimen A: Omnitrope sc dose into the abdomen of 1 mg plus a nasal dose of 100 μL CriticalSorb vehicle, administered from an Aptar UDS liquid nasal device.
- Regimen B: CP024 CriticalSorb powder prototype nasal formulation 1 (CP024-046A), containing 5 mg hGH in a 50-mg dose, administered as a single spray in each nostril using an Aptar UDS powder device.
- Regimen C: CP024 CriticalSorb powder prototype nasal formulation 2 (CP024-046B), containing 5 mg hGH in a 50-mg dose, administered as a single spray in each nostril using an Aptar UDS powder device.
- Regimen D: hGH nasal solution formulation (without CriticalSorb), containing 5 mg hGH in a 100-μL dose, administered from an Aptar UDS liquid nasal device.
- Regimen E: CP024 CriticalSorb powder prototype nasal formulation 2, containing 5 mg hGH in a 50-mg dose, administered as a single spray in each nostril twice a day using an Aptar UDS powder device, the two dosing occasions separated by 7 hours.

### Subjects

The study subjects were healthy male volunteers aged 22–50 years, with a body mass index of 18.5–30 kg/m^2^ or, if outside the range, considered not clinically significant by the investigator.

Subjects were excluded from the study for a specified number of reasons, the most significant of which were if they had a history of any drug or alcohol abuse in the past 2 years; if the regular alcohol consumption in males was equivalent to more than 210 mL pure alcohol per week; if they had smoked within the last 12 month; if a clinically significant abnormal biochemistry, hematology, or urinalysis was found as judged by the investigator; or positive hepatitis B virus, hepatitis C virus, or HIV results; and if IGF-1 levels outside the normal range as specified by the testing laboratory were found; or any nasal/respiratory structural defects or ongoing conditions.

### Laboratory data

At the screening of the volunteers, hematology, clinical chemistry, and urinalysis were performed together with testing for hepatitis B and C/HIV. Safety blood samples and urinalysis samples were taken before drug administration as a baseline and prior to discharge on each period. On admission to the clinical unit, subjects were tested for drugs of abuse, breath alcohol, and carbon monoxide. Electrocardiogram and vital signs were assessed 1 hour before dosing and at 1, 2, 4, 12 and 24 hours after the dose. Blood glucose measurements were assessed at −0.75 and −0.5 hours during the octreotide infusion and then at 0, 0.5, 1, 1.5, 2, 3, 4, 6, 8, 10, 12, 16, 18, and 24 hours after the dose.

### Pharmacokinetics (PK) and pharmacodynamics (PD)

In all periods of testing, the endogenous GH secretion was suppressed by an iv infusion of 40 μg/h octreotide commencing 1 hour before the dosing of the reference or prototype hGH product and ending upon sampling the last plasma sample, ensuring that the infusion covered the period of PK and PD sampling. Blood samples were taken at various time points throughout the study as follows below.

### Sample collection

Venous blood samples were withdrawn via an in-dwelling cannula or by venipuncture for analysis of hGH and IGF-1 at various time points before and after the dose. For regimens A to D, serum hGH levels after both nasal and sc dosing were measured −1 hour (during the octreotide infusion), 0 (before the dose), and approximately 5, 15, 30, and 45 minutes after dosing followed by bleeds at 1, 2, 3, 4, 6, 8, 10, 12, and 24 h after the dose. IGF-1 concentrations were measured before the dose at 30 minutes and 1, 3, 6, 12, and 24 hours after each dose. For regimen E the following time schedule was used for hGH sampling: for the first dose: −1 hour (during octreotide infusion), 0 (before the dose), and at 5, 15, 30, and 45 minutes and 1.5, 2, 3, 4, 5, 6, and 7 hours after the dose. For the second dose the following time schedule was used: before the dose; 30, 15, 30, and 45 minutes; and 1.5, 2, 3, 4, 5, 6, 7, 8 10, 12, 18, and 24 hours after the dose. IGF-1 concentrations were measured for the first dose at predose 30 minutes, 1.5, 3, 6, and 7 hours after the dose and the second dose at 30 minutes and 1.5, 3, 6, 10, 12, 18, and 24 hours after the dose.

### Bioanalytical methods

Blood samples were collected into serum separating tubes, left to clot at room temperature; the serum was collected after centrifugation at 3000 rpm for 10 minutes at room temperature and frozen at −70°C prior to analysis. Plasma hGH and IGF-1 levels were determined by ELISAs from R&D Systems (product numbers DGH100 and DG100), which were validated prior to use.

### Pharmacokinetic analysis

PK and PD assessments were performed by a noncompartmental analysis of the PK data using industry standard software (WinNonLin version 5.1; Pharsight Corp) (28).

### Statistical methods

The analysis of the PK data for bioavailability of nasal hGH relative to the sc injection was assessed using the following PK parameters: maximum serum concentration (C_max_), area under the curve (AUC)_0–2h_, AUC_0–7h_, and AUC_0-last_. The analysis of the PD data for IGF-1 concentrations was assessed using an ANOVA and a *t* test. The minimum serum concentration and C_max_ of regimen D was compared against regimens A, B, C, and E.

## Results

### Subject demographics

Eight white male subjects were enrolled in the study (mean age 38.1 y). The mean BMI was 26.50 kg/m^2^. No subjects withdrew from the study. All subjects who were dosed with CP024 were included in the PK and PD analysis and the safety and tolerability evaluation.

### Safety

During the study, no serious adverse events (AEs) were reported, and no subject was withdrawn from the study due to the treatments. A total of nine AEs were assessed as due to local nasal irritation and related to CP024, all of which were transient and were assessed as mild in severity (Table 1). Of these, only one subject reported five of the AEs and overall the most common were rhinorrhea and sensation of pressure in the nose (three AEs reported by two subjects for each). There were no clinically significant findings in the clinical laboratory measurements, vital signs assessments, electrocardiogram recordings, or physical examinations.

**Table 1.** Incidence of Nasal Irritation Events Considered Probably Related to CP024

Regimen hGH Dose/Route, n	A sc hGH (n = 8)	B 5 mg/IN GH+CS (n = 8)	C 5 mg/IN GH+CS (n = 8)	D 5 mg/IN GH-CS (n = 8)	E 10 mg/IN GH+CS B.D (n = 8)
Total number of subjects reporting IMP-related local nasal irritation AEs	0	2	2	0	4
Total number IMP-related local nasal irritation AEs	0	2	2	0	6
Sneezing	0	0	0	0	2
Rhinorrhea	0	0	0	0	3
Sensation of pressure in the nose	0	1	1	0	1
Burning sensation	0	1	1	0	0

IN, intranasal; CS, CriticalSorb; IMP, Investigational Medicinal Product.

### Pharmacokinetics

**Figure 1.**
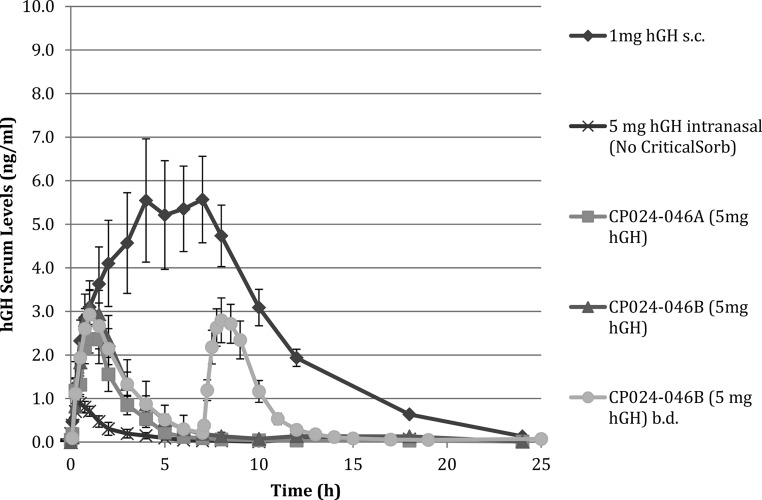
hGH Serum levels after administration of 1 mg hGH sc compared with intranasal CP024 containing 5 mg hGH once or twice daily and intranasal hGH administered without the enhancer control. Data represent the mean hGH serum concentrations of eight volunteers ± SEM.

CP024 was administered as two different formulations (A and B) at a dose of 5 mg per nostril once daily compared with the same hGH dose administered nasally as a simple solution (without the CriticalSorb absorption enhancer) and 1 mg hGH sc. CP024 B was also administered twice a day separated by 7 hours. The 7-hour separation between doses was selected on the basis of the PK and PD profiles obtained in regimens B and C. Figure 1 shows the resulting PK profiles and Table 2 the corresponding PK parameters. On at least 10 occasions, a reflux of powder was seen emitted out of the nostrils on dosing and hence in these volunteers the nasal formulation was not fully delivered into the nasal cavity. Therefore, the relative bioavailability of CP024 is likely to be an underestimate of the true bioavailability.

**Table 2.** hGH PK Parameters (Geometric Mean and Geometric Coefficient of Variations Values Unless Otherwise Stated)

Regimen hGH Dose/Route, n	A 1 mg, sc (n = 8)	B 5 mg/IN GH+CS (n = 8)	C 5 mg/IN GH+CS (n = 8)	D 5 mg/IN GH-CS (n = 8)	E 5 mg/IN GH+CS Dose 1 (n = 8)	E 5 mg/IN GH+CS Dose 2 (n = 8)	E 10 mg/IN GH+CS Dose 1+2 (n = 8)
C_max_, pg/mL	5780 (55.1)	2510 (46.0)	2810 (52.8)	911 (48.7)	2850 (66.2)	2750 (45.4)	3530 (32.5)
T_max_, h^a^	5.000 (4.00–8.00)	1.500 (1.02–2.00)	1.010 (0.75–1.50)	0.520 (0.50–1.00)	1.010 (0.50–4.00)	1.255 (0.75–2.00)^b^	1.010 (0.50–9.03)
AUC_0–2 h_, pg/h · mL	5010 (51.7)	3160 (47.9)	4000 (55.9)	1190 (52.3)	3660 (85.2)	NC	NC
AUC_0–7 h_, pg/h · mL	27 700 (50.2)	NC	NC	1860 (46.7)	7480 (66.2)	NC	NC
AUC_0-last_, pg/h · mL	55 600 (37.2)	7480 (45.9)	9380 (58.9)	2890 (27.2)	NC	8230 (45.8)	16 800 (32.7)
AUC_0-inf_, pg/h · mL	56 300 (36.4)	NC	NC	NC	NC	NC	NC
t_½_, h	3.219 (24.7)	NC	NC	NC	NC	NC	NC
F_rel0–2 h_ (reference A)	NC	0.132 (43.8)	0.157 (51.9)	0.046 (65.3)	0.137 (121.3)	NC	NC
F_rel0–2 h_ (reference D)	NC	2.895 (70.8)	3.460 (54.3)	NC	3.018 (88.0)	NC	NC
F_rel0-last_ (reference A)	NC	0.028 (47.3)	0.033 (53.9)	0.010 (40.2)	NC	0.028 (62.8)	0.029 (46.9)
F_rel0-last_ (reference D)	NC	2.817 (43.7)	3.335 (54.7)	NC	NC	2.824 (59.3)	2.857 (45.7)

Abbreviations: AUC_0–2 h_, AUC 0–2 hours; AUC_0–7 h_, AUC 0–7 hours; AUC_0-last_, AUC 0-final time point; AUC_0-inf_, AUC 0 to infinity; F, bioavailability; NC, not calculated; reference A, with reference to regimen A; reference D, with reference to regimen D; T½, half-life; IN, Intranasal; CS, CriticalSorb.

a Median (range) for t_max_ only.

b Time relative to second dose.

After the sc administration of 1 mg hGH, the median time to maximum serum concentration (T_max_) was 5.0 hours and terminal half-life 3.2 hours, which are similar to the reported values for Omnitrope (reference summary of product characteristics). hGH administered intranasally in a simple solution formulation was not absorbed into the blood stream to a great extent, with a bioavailability of approximately 1% relative to a sc injection and C_max_ at 0.9 ng/mL as shown by regimen D. However, after the nasal administration of both CP024 A and B, approximately 3 times more hGH was absorbed into the blood stream, with a similar rate of absorption to that found after the sc injection but with a shorter T_max_ (CP024 T_max_ = 1.0 h). The variability in the pharmacokinetics after the intranasal administration of hGH was similar to that observed after the sc injection (the C_max_ coefficients of variation were 55.1%, 46.0%, and 52.8% for SC hGH, and CP024 once or twice daily, respectively). When CP024 B was dosed twice daily, the PK profile of CP024 after the first dose was similar to the second dose as well as similar to the single-dose administration regimen. This is important because it indicates that on repeated dosing, pharmacokinetics and consequently PD will be reproducible.

### Pharmacodynamics

**Figure 2.**
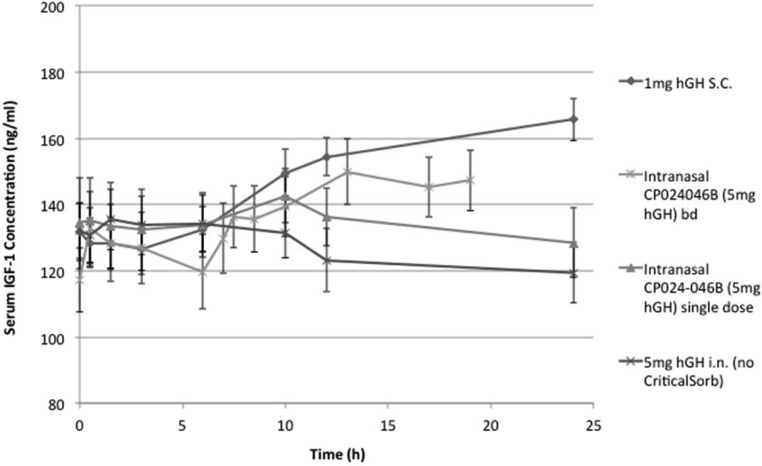
IGF-1 levels with time after administration of CP024 once and twice daily compared with sc injection and intranasal hGH control.

Three hours after the sc injection of hGH, there was a significant (*P* < .003) induction of IGF-1 that was sustained up to the 24-hour time point (Figure 2). The intranasal hGH control formulated without the CriticalSorb absorption enhancer had no effect on IGF-1 levels. However, a single dose of CP024 induced a significant increase in IGF-1 levels between 3 and 10 hours after dosing (*P* < .001). There was no significant difference between the IGF-1 levels achieved after a sc injection and CP024 at 10 hours after the dosing. Between 10 and 24 hours after a single dose of CP024, the IGF-1 levels slowly declined to baseline. When CP024 was dosed twice daily, it strongly and significantly induced IGF-1 up to at least 19 hours after the first dose (*P* < .002), achieving a similar IGF-1 C_max_ and AUC_0–10h_ to the sc injection.

**Figure 3.**
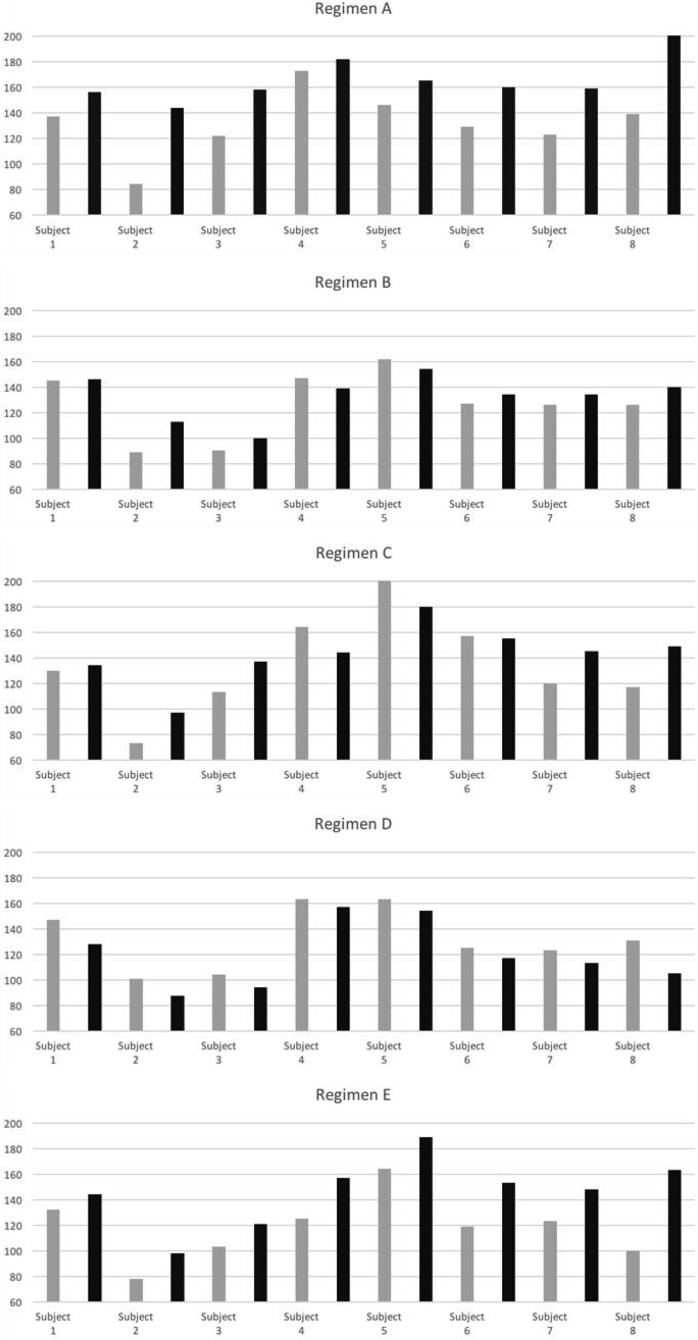
Difference between the minimum IGF-1 concentration at start of treatment (shaded bar) and maximum IGF-1 concentrations after treatment (solid bar) for each subject after treatment by regimens A–E (x-axis IGF-1 concentration [nanograms per milliliter]). No significant difference between regimens A and E (*P* = .619) were found. Regimens A and E were significantly different from regimen D (*P* = .0001).

For each volunteer the difference in the minimum IGF-1 concentration at the start of treatment) and C_max_ (maximum IGF-1 concentration after treatment) was determined and the values plotted in Figure 3 for each volunteer for each treatment regimen. When hGH was administered nasally without CriticalSorb, no IGF-1 inductions occurred and levels continually fell throughout the 24-hour period, as shown by the fall in IGF-1 concentrations. There was no significant difference in IGF-1 elevation when hGH was administered sc or as a twice-daily nasal spray (*P* = .55). The elevation in IGF-1 serum concentrations for each subject when treated with either sc dose of hGH or CP024 as a biodose was highly significant (*P* = .03) compared with the intranasal administration of hGH alone without an absorption promoter.

## Discussion

This study investigated the safety, PK, and PD of a new nasal formulation of hGH, compared with a sc injection. The CriticalSorb absorption vehicle and hGH nasal formulations were well tolerated, with any local nasal irritation being mild and transient. As a nasal spray, the nasal administration of hGH has the advantage of not having side effects associated with hGH injections, including pain, injection site reactions, inflammation, bruising, and lipoatrophy. Lack of pain, needle free, and safety all scored high in patient surveys (29, 30), and nasal delivery of hGH is therefore likely to be preferred by patients. Previous attempts to deliver hGH intranasally reported that the formulations were less well tolerated with frequent reports of pain, itching, burning, stinging, and an unpleasant taste (4, 20, 22, 31). These side effects almost certainly resulted from the absorption enhancers used (sodium taurodihydrofusidate and dipalmitoyl phosphatidylcholine), causing irritation.

The formulation was able to significantly increase the bioavailability of nasally administered hGH, but it is likely that the bioavailability calculations are a low estimate of the actual value due to the blowback/powder loss effect observed with the Aptar Pharma unit powder dose device, the prolonged absorption from the sc dose, and potentially a loss of the nasally administered hGH via the nasopharynx into the stomach in which it would be degraded and digested.

Importantly, the nasal hGH formulation containing CriticalSorb also induced IGF-1, with the levels obtained after twice-daily nasal dosing being the same (not significantly different) as for a sc injection of the hGH up to 19 hours after the first administration. The 7-hour interval between doses for regimen E was chosen based on the evaluation of the PK/PD data from regimens B and C to give the optimal rise in IGF-1 concentrations over a 24-hour period. This is the first report of IGF-1 induction in man after the intranasal administration of hGH. For example, Laursen et al (4) reported the bioavailability and bioactivity of nasal GH (0.05, 0.10, 0.20 IU/kg) in GH-deficient patients and found that only the sc injection of the hormone induced increased levels of IGF-1 in the patients. Intriguingly, given the lower hGH exposure observed after dosing with CP024 compared with the sc injection, it appears that for a given plasma level of hGH, intranasal dosing results in a greater induction of IGF-1 compared with a sc injection. This could be due to the hGH plasma profile more closely resembling the endogenous pattern of GH secretion in healthy individuals.

Endogenous GH is stored and secreted from the anterior pituitary gland in a pulsatile fashion throughout the day, each burst lasting 2–4 hours and the largest peak occurring just after the onset of sleep (3). The pharmacokinetics observed after the nasal administration of CP024 are reminiscent of these endogenous bursts, with elevated levels lasting approximately 4 hours. The effect of pulsatile GH on the growth rate of hypophysectomized rats has been explored by different researchers using iv injections of hGH. The studies suggested that frequent GH (pulsative) administration was superior to infrequent delivery or continuous infusion in terms of promotion of bone growth or body weight (32, 33) and increased serum IGF-1 (34). It was found in GH-deficient children that a daily sc dose of hGH injected at night before sleep rather than a two- to three-times-weekly dose resulted in a significant increase in growth for the same total dose of 12 IU/wk (35). The results were found in two similar studies in which the total dose was changed from being administered three times weekly im to daily administration by sc injection (36, 37). A study evaluating the effect of a pulsatile vs continuous iv administration of hGH in GH-deficient patients (38) found that eight boluses (given at 3 h intervals starting at 8:00 pm) or a continuous infusion of a fixed total hGH dose were equally effective in generating an increase in IGF-1, whereas the same amount of hGH given as two large boluses (at 8:00 pm and 2:00 am) resulted in a significantly smaller increase in IGF-1.

Serum IGF-1 levels have been correlated with the achieved growth rate after GH replacement therapy (39, 40). Achieving the same IGF-1 induction with lower serum GH levels has the potential to maintain efficacy of treatment while reducing the risk of side effects due to the direct actions of hGH. In particular, given the increasing incidence of diabetes, there is increasing concern of GH's effects on insulin sensitivity (41). Indeed, an analysis of data from the Pharmacia KIGS postmarketing surveillance database found an increased incidence of diabetes in children treated with GH replacement therapy (42). By achieving similar IGF-1 levels with lower systemic exposure to hGH, CP024 may mitigate this risk.

In conclusion, we have developed a nasal spray formulation of hGH able to induce IGF-1 levels to a similar extent as that observed after sc injection. Because nasal sprays are pain free and relatively simple to administer, CP024 could be an attractive treatment option for patients, particularly children and needle-phobic patients, but in addition, because a similar PD effect is possible with lower systemic hGH exposure, there could be less potential for insulin insensitivity.
